# Connecting the dots: approaching a standardized nomenclature for molecular connectivity in positron emission tomography

**DOI:** 10.1007/s00259-025-07357-1

**Published:** 2025-06-02

**Authors:** Murray B. Reed, Luca Cocchi, Christin Y. Sander, Jingyuan Chen, Granville J. Matheson, Patrick Fisher, Tommaso Volpi, Nikkita Khattar, Christine DeLorenzo, Gregor Gryglewski, Leo R. Silberbauer, Matej Murgaš, Godber M. Godbersen, Lukas Nics, Martin Walter, Marcus Hacker, Alessandra Bertoldo, Mark Lubberink, Mark Silfstein, R. Todd Ogden, J. John Mann, Tetsuya Suhara, Andrea Varrone, Ronald Boellaard, Roger N. Gunn, Alexander Hammers, Bharat Biswal, Bruce Rosen, Gitte M. Knudsen, Richard Carson, Julie Price, Rupert Lanzenberger, Andreas Hahn

**Affiliations:** 1 Department of Psychiatry and Psychotherapy, Medical University of Vienna, Vienna, Austria; 2 Comprehensive Center for Clinical Neurosciences and Mental Health (C3 NMH), Medical University of Vienna, Vienna, Austria; 3 Department of Mental Health and Neuroscience, QIMR Berghofer Medical Research Institute, Brisbane, 4006 Australia; 4 Athinoula A. Martinos Center for Biomedical Imaging, Department of Radiology, Massachusetts General Hospital and Harvard Medical School, Massachusetts General Hospital A.A. Martinos Center for Biomedical Imaging, Boston, MA USA; 5 Department of Clinical Neuroscience, Center for Psychiatry Research, Karolinska Institutet and Stockholm County Council, 171 76 Stockholm, Sweden; 6 Neurobiology Research Unit, Copenhagen University Hospital Rigshospitalet, Copenhagen, Denmark; 7 Department of Clinical Medicine, Faculty of Health and Medical Sciences, University of Copenhagen, Copenhagen, Denmark; 8 Department of Radiology and Biomedical Imaging, Yale University, New Haven, CT USA; 9 Department of Psychiatry and Behavioral Health, Stony Brook University, Stony Brook, NY USA; 10 Department of Biomedical Engineering, Stony Brook University, Stony Brook, NY USA; 11 Child Study Center, Yale University, New Haven, CT USA; 12 Department of Biomedical Imaging and Image-Guided Therapy, Division of Nuclear Medicine, Medical University of Vienna, Vienna, Austria; 13 Department of Psychiatry and Psychotherapy, Jena University Hospital, Jena, Germany; 14 Clinical Affective Neuroimaging Laboratory (CANLAB), Otto-Von-Guericke-University Magdeburg, 39120 Magdeburg, Germany; 15 Department of Behavioral Neurology, Leibniz Institute for Neurobiology, 39118 Magdeburg, Germany; 16 Center of Behavioral Brain Sciences, Otto-Von-Guericke University, 39118 Magdeburg, Germany; 17 Padova Neuroscience Center, University of Padova, Padova, Italy; 18 Department of Information Engineering, University of Padova, Padova, Italy; 19 Nuclear Medicine and PET, Department of Surgical Sciences, Uppsala University, Uppsala, Sweden; 20 Irving Medical Center Departments of Biostatistics, Columbia University, New York, NY USA; 21 Columbia University Irving Medical Center Departments of Psychiatry and Radiology, and New York State Psychiatric Institute, New York, NY USA; 22 National Institutes for Quantum Science and Technology, Anagawa, Inage-Ku, Chiba, Japan; 23 Department of Radiology & Nuclear Medicine, Amsterdam UMC, Amsterdam, The Netherlands; 24 Department of Nuclear Medicine and Molecular Imaging, University Medical Center Groningen, Groningen, The Netherlands; 25 Xing Imaging– A Mitro Company, London, UK; 26 Brain Sciences, Imperial College London, Hammersmith Hospital, London, UK; 27 King’s College London & Guy’s and St Thomas’ PET Centre, London, UK; 28 School of Biomedical Engineering & Imaging Sciences, King’s College London, London, UK; 29 Department of Biomedical Engineering, New Jersey Institute of Technology, Newark, NJ USA

**Keywords:** Metabolic connectivity, Molecular covariance, Functional PET (fPET), Consensus, Terminology

## Abstract

Positron emission tomography (PET)-based connectivity analysis provides a molecular perspective that complements fMRI-derived functional connectivity. However, lack of standardized terminology and diverse methodologies in PET connectivity studies has resulted in inconsistencies, complicating the interpretation and comparison of results across studies. A standardized nomenclature is thus needed to reduce ambiguity, enhance reproducibility, and facilitate interpretability across radiotracers, imaging modalities and studies. Here, we define and differentiate the terms “molecular connectivity” and “molecular covariance”. Drawing parallels from other imaging modalities, we propose “molecular connectivity” as an umbrella term to characterize statistical dependencies between the measured PET signal across brain regions at a *within-subject* level. Like fMRI resting-state functional connectivity, “molecular connectivity” leverages spatio-temporal associations in the PET signal to derive brain network associations. Conversely, “molecular covariance” denotes group-level computations of covariance matrices *between-subjects*. Further specification of the terminology can be achieved by including the target of the employed radioligand, such as “metabolic connectivity/covariance” for [^18^F]FDG or “amyloid covariance” for [^18^F]flutemetamol and “tau covariance” for [^18^F]flortaucipir. While this approach to standardization aims to clarify terminology, open questions remain about the neurobiological underpinnings of these connectivity metrics. Future research should focus on elucidating these mechanisms and developing advanced computational methodologies that evaluate diverse feature relationships and improve the robustness of PET-based connectivity metrics.

**Supplementary Information:**

The online version contains supplementary material available at 10.1007/s00259-025-07357-1.

## Introduction

The assessment of resting-state functional brain networks, as mostly elucidated through functional Magnetic Resonance Imaging (fMRI) and Electroencephalography (EEG), has been a cornerstone of neuroimaging research for decades due to its low cost, widely available hardware and software and low risk due to its non-invasiveness. Functional connectivity (FC) derived from both resting-state and task activity have provided valuable insights into the organization of the brain and network interactions by correlating moment-to-moment fluctuations of signals between spatially distinct brain regions [1]. Positron Emission Tomography (PET) enables imaging of molecular and biochemical processes and can quantify energy metabolism, receptor binding, enzyme activity, and other molecular targets at nanomolar concentrations. However, connectivity analysis of PET data remains relatively unexplored, whereas initial studies have already highlighted differences in metabolic and functional connectivity patterns [2–4]. As such, molecular connectivity offers a complementary perspective on physiological processes, distinct from the functional connectivity observed in fMRI and EEG/MEG studies. While molecular connectivity is a concept dating back to the 1980s [5] and 1990s [6, 7], little progress has been made, in part due to technological constraints in PET imaging that resulted in limited count rates at high temporal resolutions, and consequently too much noise in data collected over shorter periods. These constraints precluded the reconstruction of dynamic PET data in the range of seconds and thus the estimation of connectivity at the individual level.

**Fig. 1:**
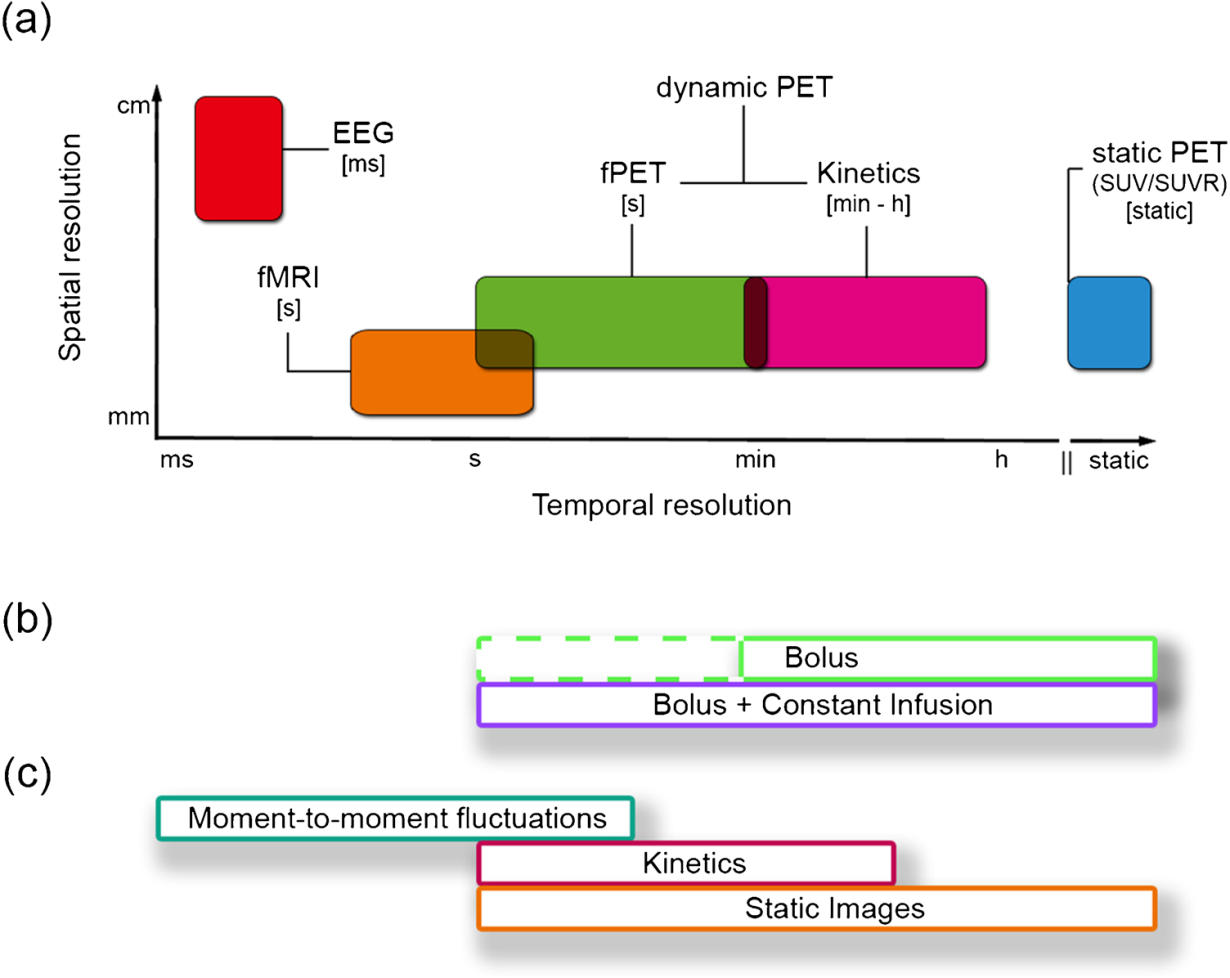
Graphical overview of common neurophysiological techniques used to assess brain connectivity. (**a**) Acquisition: Each box represents a common imaging method where the position on the x-axis indicates the temporal resolution of each technique as an input for different connectivity measures, whereas position on the y-axis represents the spatial resolution of the technique. (**b**) Radiotracer administration: In the field of PET imaging, application of the radiotracer as bolus enables to obtain static images and tracer kinetics, while a bolus + constant infusion protocol additionally allows to capture moment-to-moment signal fluctuations. In theory, moment-to-moment fluctuations may also be obtained after a bolus application, however, the rationale for bolus + infusion protocol is to provide free radiotracer throughout the scan, which then binds according to such rapid fluctuations (dashed green line) (**c**) Input for connectivity estimation: dynamic approaches enable the computation of within-subject connectivity, which is typically calculated by correlating the time courses among brain regions. For PET this is derived from either bolus plus constant infusion or simple bolus. In contrast, between-subject covariance is estimated over a group of participants as it lacks a temporal component. As such, these techniques use different input signals to estimate connectivity, i.e., moment-to-moment fluctuations in the signal or radiotracer kinetics for within-subject metrics, and covariance of PET outcome measures for between-subjects PET signal covariance (i.e., static images). Furthermore, static images can also be obtained from dynamic PET data, e.g., through kinetic modeling, and subsequently enable estimation of covariance metrics. EEG: electroencephalography, fMRI: functional magnetic resonance imaging, fPET: functional positron emission tomography, SUV(R): standardized uptake value (ratio)

Consequently, the computation of correlations across subjects became the dominant approach as a proxy for molecular connectivity (often termed “covariance matrix”, although not referring to the traditional statistical inference framework). In this context, the widespread availability of [^18^F]Fluorodeoxyglucose ([^18^F]FDG) for metabolic connectivity (i.e., molecular connectivity for glucose metabolism), represents a promising avenue to move the field forward by probing brain interactions based on metabolic demands, complementing blood-oxygen-level-dependent (BOLD) response, its fMRI analogue [8–12]. However, the inherent disadvantage of estimating associations across an entire group of subjects instead of connectivity at the subject level considerably limits its individual biological interpretation [2, 13]. Figure 1 presents a graphical overview of common techniques used to assess brain connectivity in humans in vivo*,* highlighting the relationship of PET to other imaging modalities.

Recent technological progress has transformed the landscape of molecular connectivity research. With increased sensitivity of PET scanners, specific infusion protocols (i.e., bolus + constant infusion), refined reconstruction algorithms, and advanced pre-processing including filtering techniques and post-processing, researchers are now better equipped to investigate brain networks at a molecular level. These advances enable the use of PET data at unprecedented temporal resolutions, within the range of minutes and seconds [2, 14, 15], approaching the temporal dynamics of fMRI [14]. These developments have established the foundation for estimating individual temporal molecular connectivity through various computational methodologies. Techniques include the application of within-subject Euclidean distance metrics [16], signal detrending by third-order polynomial functions [17, 18], spatiotemporal filtering [2], baseline normalization [19], and potentially, band-pass filtering, as commonly performed in fMRI [20]. These approaches aim to infer individual-level connectivity by using temporal information derived from PET data. In most cases, this requires the removal of the baseline tracer uptake from the PET signal, which is achieved by filtering or normalization. The residual signal allows the correlation of moment-to-moment fluctuations in the PET signal. On the other hand, the Euclidean distance method evaluates similarities in the raw time-activity curves (TACs) or individual compartmental time courses derived from kinetic modeling. In contrast, between-subject approaches mostly use covariance matrices [21–24] or sparse inverse covariance estimation (SICE) [21], which compute associations between brain regions across subjects. Similar to molecular connectivity, covariance matrices across groups can also be estimated using principal (PCA) or independent component analysis (ICA), as shown previously [12, 25–27]. Although these may yield loadings for individual subjects, they still require an entire group for the estimation.

Finally, various hybrid approaches have been introduced, which integrate fMRI and PET metrics [28, 29]. The core challenge is that each of these techniques has been labelled as “molecular connectivity”, despite differences in the underlying assumptions, computations, and outcome metrics, resulting in inconsistent terminology. Moreover, related terms such as “metabolic connectivity mapping” are employed in different contexts to describe various outcomes, leading to potential confusion [16, 28]. The multimodal approach utilizes both a static PET image and fMRI data to determine directional connectivity [28]. On the other hand, the PET-only approach utilizes only the raw TACs from PET images and is the equivalent to our proposed eMC term.

As the field of PET-based connectivity (research and clinical translation) experiences a rapid growth in utilization and methodological diversity (Supplementary Figure 1), there is a pressing need for standardization in nomenclature. The absence of a unified terminology poses challenges for synthesizing findings across studies and impedes the development of a cohesive framework for interpreting PET connectivity outcomes. For example, some studies have applied covariance-based analyses to within-subject data, while others have used the same terminology for group-level comparisons (see Supplementary Table 1), making cross-study comparisons difficult due to inflated correlation values. Of note, these concepts could similarly be applied to SPECT imaging. Discussions regarding the definition of molecular connectivity and covariance, as well as the distinct, yet valuable insights offered by each approach, have already commenced [2, 13, 23]. However, previous work either compared only a subset of approaches, or was qualitative in nature, while widespread consensus grounded in the actual outcome parameters of each technique is lacking.

To address this gap, we propose an approach to nomenclature standardization for the most commonly used PET connectivity techniques, namely molecular connectivity and molecular covariance. The proposed nomenclature is derived from a comprehensive review of the literature on various methodologies used to compute connectivity and covariance metrics. By integrating theoretical foundations, we seek to establish a cohesive and robust framework for defining molecular connectivity metrics. This process involved multiple online discussions among multidisciplinary and international group of researchers in the field and an in-person meeting at the Neuroreceptor Mapping 2024 conference in Montréal, Canada. The final nomenclature was refined through manuscript revisions, with all coauthors actively contributing. Similar to the standardization of nomenclature for quantification of PET radioligands [30], this effort is an important step toward improving consistency in the communication of molecular connectivity research findings. It provides the opportunity to spark further discussions with other groups and colleagues, with the goal of fostering consensus on nomenclature in the evolving field of PET-based connectivity.

## Nomenclature of molecular connectivity and covariance

Given the growth of interest in PET connectivity research in recent years (see Supplementary Figure 1), the standardization of its nomenclature is fundamental to address several pivotal issues within the field. One major issue is the inconsistent use of terms, where a single term may describe multiple distinct concepts (and vice versa). This ambiguity complicates the interpretation of outcomes across studies, making it unclear what is being measured or reported. Harmonizing the terminology can facilitate unambiguous communication increasing the reproducibility of findings across studies. A standardized nomenclature also enhances comprehension among diverse audiences (e.g. neuroscientists, psychiatrists, neurologists) within broader contexts (research and clinical) and diverse levels of expertise, including non-imaging experts. Standardized terminology enables researchers from disparate backgrounds to readily compare findings, fostering knowledge exchange and interdisciplinary cooperation.

**Fig. 2:**
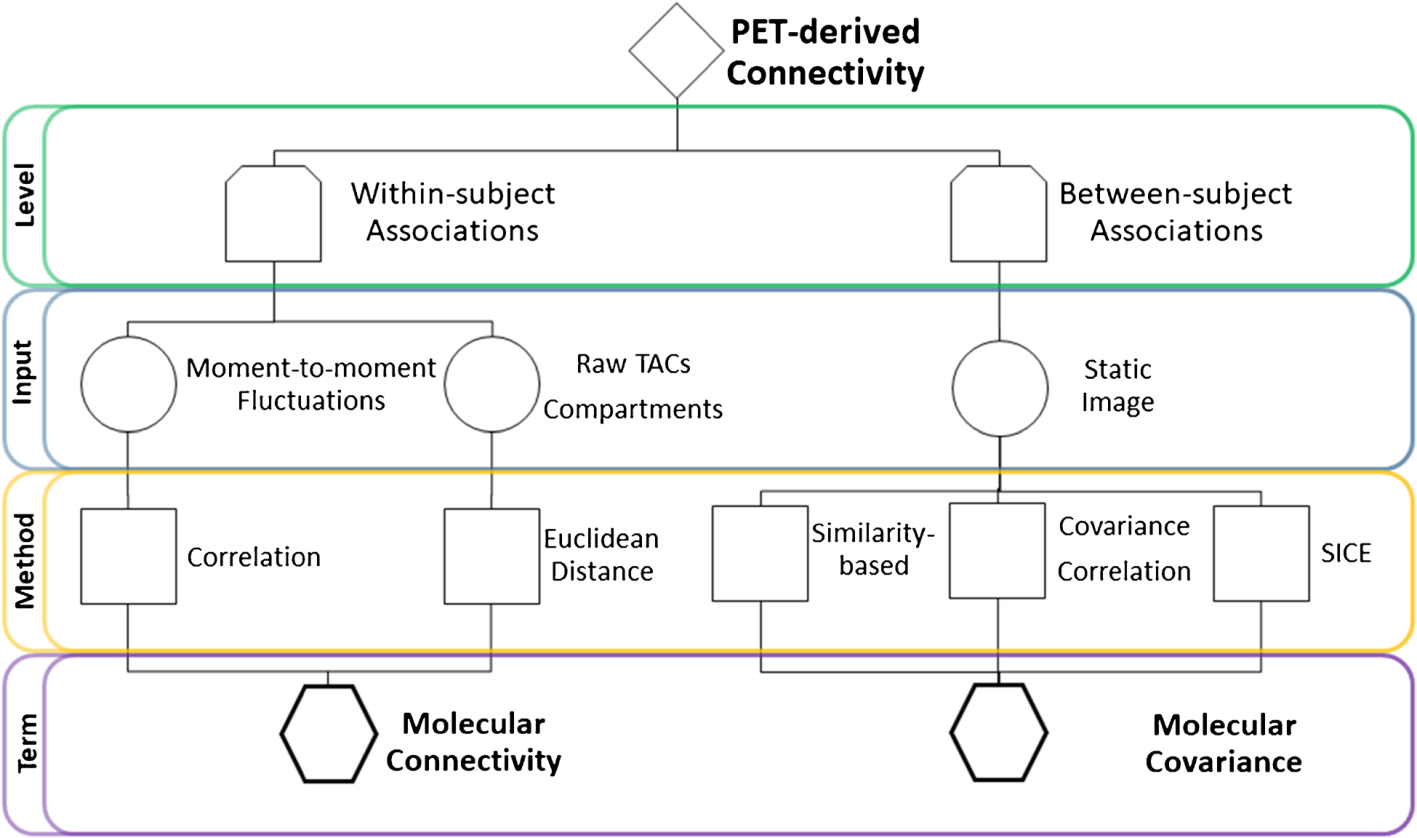
Summary of techniques and proposed nomenclature of PET-based connectivity. Within-subject connectivity is broadly separated into the assessment of moment-to-moment signal fluctuations and raw time activity curves (TACs) or compartment time courses. The former requires the elimination of the low-frequency baseline radiotracer uptake and reducing high-frequency noise, which can be achieved using different detrending methods. Similar to fMRI-based functional connectivity, the correlation of moment-to-moment signal fluctuations are used to estimate correlationed-based “molecular connectivity” (cMC). The Euclidean distance can be used to estimate similarities using either raw TACs or radiotracer compartment time courses between brain regions, thus termed Eucledean-based “molecular connectivity” (eMC). The between-subject metrics use any type of static PET image as input and after computation using methods like covariance/correlation or SICE, are termed as “molecular covariance” (mCov). Moreover, within the mCov term, similarity-based methods that utilize static images to define network relationships using metrics such as cosine similarity, inverse Euclidean distance, or graph-based measures are also included

Historically, PET connectivity methodologies have been grouped under the umbrella term “molecular connectivity” (and with the common use of [^18^F]FDG to obtain “metabolic connectivity”) as a concept encompassing both within-subject connectivity and between-subject analyses. However, the interchangeable use of “molecular connectivity” for within-subject and between-subject associations is problematic, as these represent fundamentally distinct measures which are used for different purposes. Generally, most imaging modalities use “connectivity” to characterize the strength (and potential directionality) of correlations between brain regions *within subjects* (Fig. 1). This is true for functional connectivity obtained from fMRI and EEG data and structural connectivity derived from diffusion-weighted MRI, regardless of whether the calculations are data-driven or seed-based. On the other hand, “covariance” metrics gauge the statistical associations between regions of interest of a static outcome metric *between* individuals, such as gray matter volume obtained from T_1_-weighted structural images [31, 32] or Standardized Uptake Value Ratio (SUVR) [33], binding potential maps, etc. in PET imaging. While the distinction is typically specified once in the methods section, the term “molecular connectivity” is often used throughout the remainder of the manuscript without further clarification. As a result, the critical distinction between within-subject and between-subject associations tends to be lost in interpretation, underscoring the need for more standardized and precise terminology to maintain clarity. Recent studies in structural MRI similarity networks [34, 35] suggest that similarity-based approaches could complement existing PET connectivity frameworks. Similarity-based methods quantify molecular covariance by assessing interregional similarity in tracer uptake or kinetic parameters rather than temporal correlations or statistical covariance. A clear differentiation between these concepts is imperative to prevent misinterpretation and to ensure accurate communication of study findings (Fig. 2).

For “molecular connectivity” analyses, we therefore suggest that this term encompass all approaches that use moment-to-moment fluctuations in the measured PET signal to estimate connectivity (like “functional connectivity” used in fMRI). In this context, further detail emerges for *within-subject* connectivity methods, each capturing temporal dynamics within an individual, contingent upon the method employed to estimate the connectivity metrics. On the other hand, “molecular covariance” should be used to estimate network interactions *between-subjects*, i.e., when an entire group of subjects is required for the estimation. The discourse on the disparities between molecular connectivity and molecular covariance is not novel [13, 36, 37], and will therefore not be repeated here. These discussions underscore the relevance of *within-subject* and *between-subject* statistical metrics in PET connectivity research and their inherent differences. While within-subject connectivity furnishes insights into individual variability and dynamic network properties, between-subject covariance offers a broader perspective on shared patterns of brain activity across populations. Unlike prior discussions, which mainly describe these differences [13, 37], our manuscript offers a solution to the inconsistency problem by providing a clear framework for terminology that can be applied across studies.

Another molecular connectivity approach, different from the former methods is the Euclidean distance calculation. This divergence stems from the diversity of inputs it accommodates (raw or compartment-specific TACs) and how connectivity is calculated (Euclidean distance between TACs). In this regard, the method is theoretically limited to estimating solely positive connectivity metrics, as it relies on calculating the absolute Euclidean distance between two TACs. Nevertheless, the approach aims to derive connectivity metrics within an individual and is thus classified under “molecular connectivity”. Notably, extension with kinetic modelling allows derivation of kinetic connectivity for the individual compartments, yielding relevant information about the transport across the blood–brain barrier (K_1_, k_2_) and irreversible uptake into the cells (k_3_). To distinguish between these two approaches to estimating "molecular connectivity," we propose the adoption of specific subterms. Molecular connectivity estimated using correlations should be referred to as"correlation-based molecular connectivity"(cMC), while connectivity based on Euclidean distance should be termed "Euclidean-based molecular connectivity"(eMC). Table 1 provides an overview of these proposed terms and their definitions. For recent use cases on empirical data, please refer to the following references for cMC [2, 19], eMC [3, 16], mCov (see Supplementary Table) and SICE [24], respectively.

**Table 1:** Description of each PET connectivity and covariance term and its respective abbreviation, level of estimation and definition

Term	Abbreviation	Subterm	Level	Definition
PET-based Connectivity				PET-based connectivity refers to the assessment of connections and interactions between molecular entities within the brain. It is an umbrella term that encompasses all subsequent terms
Molecular Connectivity	MC	Correlation-based molecular connectivity (cMC) or Euclidean-based molecular connectivity (eMC)	Within-subject	Molecular connectivity estimates associations between brain regions at the individual subject level, e.g. by correlation of moment-to-moment signal fluctuations. This is akin to functional connectivity obtained with fMRI. Another option is to estimate the Euclidean distance between brain regions to evaluate the similarity of raw time-activity curves or radiotracer kinetics within an individual
Molecular Covariance	MCov	Molecular Covariance	Between-subject	Molecular covariance utilize PET-derived static images (SUV/SUVR, binding potential, etc.) across subjects to estimate interregional associations within the brain. This is akin to structural covariance obtained with T1-weighted MRI

The following examples illustrate the differences between these outcomes and the types of conclusions that can be drawn from their estimation. Molecular connectivity has predominantly been explored in the context of metabolic connectivity, largely due to the focus of existing studies, tracer availability and the ability to reconstruct high-temporal resolution data with sufficient signal to noise ratio (SNR). However, it is anticipated that next-generation PET systems will enable the examination of dynamic neurotransmitter release, measured as neurotransmitter flux across different brain regions. It is important to note that the temporal resolution of PET imaging limits its capacity to measure other regulatory mechanisms, such as the onset of internalization which can be rapid compared to receptor up- or downregulation which involves recycling the receptor or new receptor synthesis and placement in terminal membranes. In such cases, differences in the estimated within-subject connectivity metrics can be interpreted as representing specific metabolic or neurotransmitter dynamics. Regarding tracer kinetics or compartment-derived connectivity, it is important to acknowledge that these measurements are influenced by multiple biological processes. For example, tracer kinetics include contributions from protein binding, blood flow, blood brain barrier transfer, ligand transporter activity out of the brain (two types of such transporters exist: (a) carrier mediated transporters, and (b) active efflux transporters like P-glycogen that carry drugs and other compounds out of the brain), non-specific binding, receptor occupancy by an endogenous ligand, and specific binding, that is actually a measure of available target binding. Only some of these can be resolved by kinetic modeling for a clear interpretation including assay of protein binding and an arterial input function. For molecular covariance, a compelling example is the use of ICA with [^11^C]PHNO imaging to separate its binding to D2 and D3 receptors at the group level [38]. This could be related to the observation that certain protein targets are expressed at consistent relative densities across the brain.

## Tracer-specific nomenclature

For different radiotracers, we propose that “molecular connectivity/covariance” serve as an umbrella term for PET-based estimation of network interactions in general. This framework allows for the specification of different radioligands by using the corresponding biological target as a prefix. Drawing examples from previous literature, this approach yields “metabolic connectivity/covariance” for [^18^F]FDG, “5-HT_1A_ covariance” for [*carbonyl*-^11^C]WAY100635 [39], “SERT covariance” for [^11^C]DASB [40] or “amyloid covariance” for [^18^F]flutemetamol (amyloid-β) and “tau covariance” for [^18^F]flortaucipir (tau) [41]. Please see Table 2 for full nomenclature of the different radioligands and Supplementary Table 1 for a list of examples. We expect that this terminology can be extended to other targets, further underlining the need for standardized terminology and assessment of the feasibility and interpretation for radioligands beyond [^18^F]FDG.

**Table 2:** Exemplary overview of proposed terms for PET connectivity and covariance terms based on radioligands. A naming convention is proposed for additional radiotracers, wherein the convention involves stating the target (prefix) followed by the utilized connectivity or covariance. It is important to note that the feasibility of estimating molecular connectivity (i.e., moment-to-moment fluctuations) for all radiotracers in this list is not yet established (marked *)

Tracer	Molecular connectivity (MC) term	Molecular covariance (MCov) term
[^18^F]FDG	Metabolic connectivity (M-MC)	Metabolic covariance (M-MCov)
[^15^O]H2O	Cerebral blood flow connectivity (CBF-MC)	Cerebral blood flow covariance (CBF-MCov)
[^18^F]FDOPA	Dopamine synthesis connectivity (DAS-MC)	Dopamine synthesis covariance (DAS-MCov)
[*carbonyl*-^11^C]WAY100635	* 5-HT_1 A_ connectivity (5-HT_1 A_-MC)	5-HT_1 A_ covariance (5-HT_1 A_-MCov)
[^11^C]DASB	* SERT connectivity (SERT-MC)	SERT covariance (SERT-MCov)
[^18^F]flutemetamol	* Amyloid beta connectivity (Abeta-MC)	Amyloid beta covariance (Abeta-MCov)
[^18^F]flortaucipir	* Tau connectivity (tau-MC)	Tau covariance (tau-MCov)

## Combination with other imaging modalities

Recently, hybrid connectivity and covariance techniques have also emerged, leveraging the complementary strengths of static PET and fMRI-based functional connectivity to delineate molecular covariance [29] or to estimate directional connectivity [28]. Another new approach has been the integration of dynamic fPET with fMRI to investigate task-related neuronal responses using ICA [42]. Extending such combined analyses to connectivity and exploring hybrid methods that integrate data from multiple modalities such as fPET, fMRI and EEG, holds promise for providing a comprehensive understanding of brain connectivity. These hybrid approaches could also enable the investigation of directional connectivity with PET (such as dynamic causal modeling for fMRI data [43]), shedding light on the causal interactions between brain regions. Such advancements could enhance our understanding of complex neural networks underlying cognition and behavior. As the field expands, establishing and adopting specific terminology will be increasingly essential. Although it is not possible to exhaustively detail all potential combinations with other imaging modalities, the current work establishes a foundational terminology and interpretation framework. Consequently, the distinction between group-level covariance and individual connectivity may also be relevant for multimodal combinations.

## Limitations and knowledge gaps

It is important to acknowledge that the underlying neurobiological basis of some of the proposed terms and concepts have yet to be fully understood. For molecular connectivity, moment-to-moment fluctuations in the PET signal may be interpreted similarly to in BOLD fMRI, where two brain regions are considered coupled if they exhibit similar temporal behavior between their time courses, i.e. a high correlation value [44]. In this context, a high correlation value may indicate temporally coupled metabolic demands for [^18^F]FDG or neurotransmitter dynamics for other radioligands. For eMC derived from compartmental time courses, the functional coupling between brain regions is assumed if their kinetic properties are similar. However, further research is needed to elucidate the underlying changes in rate constants related to observed fluctuations and similarities in the PET signal.

For molecular covariance, interpretations are limited to the group level due to the underlying computation of an association across subjects. High correlation values indicate that brain regions show similar radiotracer binding or kinetic properties within a given group, necessitating that the group represents a homogeneous sample to identify relevant pathological alterations. It is worth noting that challenges regarding the neurobiological interpretation are not unique to PET imaging but rather apply to most imaging modalities used to investigate the living human brain, including fMRI and EEG. In fact, the specificity of PET radiotracers to a specific target may allow for a more straightforward interpretation, offering valuable complementary information to other imaging techniques. Additionally, animal models will be essential for gaining a better biological interpretation of the different connectivity metrics, as they allow for controlled interventions, facilitate the use of specialized imaging protocols or scanners that may not be feasible in human studies, and allow for a more causal investigation of biological underpinnings.

As the field and its technical capabilities continue to advance, the biological mechanisms have not yet been validated for the described concepts. This is particularly true for molecular connectivity metrics (i.e. individual moment-to-moment fluctuations of PET signals) for radiotracers other than [^18^F]FDG. However, as rapid stimulation-induced changes have already been extended to other tracers, such as dopamine and serotonin synthesis [45, 46], it is anticipated that the computation of connectivity metrics may similarly become feasible for these tracers. This feasibility may be attributed to the shared physiological aspects, such as blood–brain barrier transport and subsequent enzymatic processes (e.g., as discussed in [47]). In contrast, eMC may be applicable to any radioligand, as raw TACs are evaluated. On the other hand, the estimation of covariance matrices is computationally feasible for any given radiotracer, though the interpretation of outcomes is dependent on the underlying input, like the diagnostic homogeneity within a patient group. Such interpretations might be straightforward for hypotheses based on neuroanatomical projections between brain regions [40, 48], but becomes increasingly complex for whole-brain connectivity matrices. Another important factor when estimating metabolic covariance is the potential variability introduced by differences in spatial and temporal resolution across PET systems. To ensure comparability, various harmonization techniques have been proposed in the field of PET neuroimaging including image resolution matching, partial volume correction to mitigate signal blurring effects, and cross-scanner normalization strategies. The frame length and time between tracer application and start of the static image should be closely matched to ensure both similar SNR and consistent tracer kinetics between the images.

## Outlook and conclusion

The main goal of this work was to define a common nomenclature for PET-based associations between brain regions, both at the individual and group-average levels. More specifically, we highlight the different opportunities to derive individual-level molecular connectivity and group-level molecular covariance from PET data. We emphasize that all these approaches provide valuable insights into brain function. Still these techniques require distinct terminology due to the underlying differences in the assumptions, calculations, and outcome metrics, giving rise to different interpretations and conclusions. In addition to already available use cases, future research should focus on experimental validation of PET connectivity matrices, including comparative analyses of the different connectivity and covariance methods, cross-validation with causal approaches like animal models and multi-site studies to assess reproducibility across imaging platforms. While PET-based connectivity analyses are currently predominantly applied in research settings, promising clinical studies have already been conducted, such as those by Deery et al. [4] and Vallini et al. [3], highlighting the potential clinical value. As the evaluation of moment-to-moment fluctuations in PET signals is still in its infancy, future work should aim to identify the underlying neurophysiological mechanisms. This knowledge will boost the interpretation of PET-based connectivity approaches and may aid in identifying pathophysiological processes underpinning brain disorders. Additionally, comparing brain network interactions across imaging techniques and modalities represents a promising opportunity to deepen our understanding of brain function. While exploring alternative tracers and more advanced methodologies, such as directional molecular connectivity, holds great promise for advancing knowledge of brain networks, this progress requires a clear and consistent terminology to delineate the distinct types of brain connectivity. To ensure the adoption of this terminology, collaborative efforts within the neuroimaging community are essential. This includes workshops and methodological studies to refine definitions and establish best practices for the field of PET connectivity.

## Supplementary Information

Below is the link to the electronic supplementary material.

Supplementary file1 (PDF 609 KB)

## Data Availability

Not applicable.
