# Supplementary material for: Connecting the dots: approaching a standardized nomenclature for molecular connectivity in positron emission tomography
Source: Eur J Nucl Med Mol Imaging. 2025 Jun 2;53(1):48–58. doi: 10.1007/s00259-025-07357-1 (PMC12660352; doi:10.1007/s00259-025-07357-1)
Supplement: Supplementary file 1 — Supplementary file1 (PDF 609 KB) [file 259_2025_7357_MOESM1_ESM.pdf]

Connecting the Dots: Approaching a Standardized Nomenclature for Molecular Connectivity in Positron Emission Tomography

Reed MB[1,2], Cocchi L[3], Sander CY[4], Chen J[4], Matheson GJ[5], Fisher P[6,7], Volpi T[8], Khattar N[8], DeLorenzo C[9,10], Gryglewski G[1,2,11], Silberbauer LR[1,2], Murgaš M[1,2], Godbersen GM[1,2], Nics L[12], Walter M[13,14,15,16], Hacker M[12], Bertoldo A[17,18], Lubberink M[19], Silfstein M[9], Ogden TR[20], Mann JJ[21], Suhara T[22], Varrone A[5], Boellaard R[23,24], Gunn RN[25,26], Hammers A[27], Biswal B[28], Rosen B[4], Knudsen GM[6,7], Carson R[8], Price J[4], Lanzenberger R[1,2 #], Hahn A[1,2]

[1]Department of Psychiatry and Psychotherapy, Medical University of Vienna, Austria

[2]Comprehensive Center for Clinical Neurosciences and Mental Health (C3NMH), Medical University of Vienna, Austria

[3]Department of Mental Health and Neuroscience, QIMR Berghofer Medical Research Institute, Brisbane 4006, Australia.

[4]Athinoula A. Martinos Center for Biomedical Imaging, Department of Radiology, Massachusetts General Hospital and Harvard Medical School, Massachusetts General Hospital A.A. Martinos Center for Biomedical Imaging, Boston, Massachusetts, USA

[5]Department of Clinical Neuroscience, Center for Psychiatry Research, Karolinska Institutet and Stockholm County Council, Stockholm, SE-171 76, Sweden

[6]Neurobiology Research Unit, Copenhagen University Hospital Rigshospitalet, Copenhagen, Denmark.

[7]Department of Clinical Medicine, Faculty of Health and Medical Sciences, University of Copenhagen, Copenhagen, Denmark

[8]Department of Radiology and Biomedical Imaging, Yale University, New Haven, CT, USA

[9]Department of Psychiatry and Behavioral Health, Stony Brook University, Stony Brook, NY, USA.

[10]Department of Biomedical Engineering, Stony Brook University, Stony Brook, NY, USA.

[11]Child Study Center, Yale University, New Haven, CT, USA

[12]Department of Biomedical Imaging and Image-guided Therapy, Division of Nuclear Medicine, Medical University of Vienna, Austria

[13]Department of Psychiatry and Psychotherapy, Jena University Hospital, Jena, Germany

[14]Clinical Affective Neuroimaging Laboratory (CANLAB), Otto-von-Guericke-University Magdeburg, Magdeburg, 39120, Germany.

[15]Department of Behavioral Neurology, Leibniz Institute for Neurobiology, Magdeburg, 39118, Germany.

[16]Center of Behavioral Brain Sciences, Otto-von-Guericke University, Magdeburg, 39118, Germany.

[17]Padova Neuroscience Center, University of Padova, Padova, Italy

[18]Department of Information Engineering, University of Padova, Padova, Italy.

[19]Nuclear Medicine and PET, Department of Surgical Sciences, Uppsala University, Uppsala, Sweden.

[20]Columbia University Irving Medical Center Departments of Biostatistics, New York, NY, United States.

[21]Columbia University Irving Medical Center Departments of Psychiatry and Radiology, and New York State Psychiatric Institute, New York, NY, United States.

[22]National Institutes for Quantum Science and Technology, Anagawa, Inage-ku, Chiba, Japan

[23]Department of Radiology & Nuclear Medicine, Amsterdam UMC, Amsterdam, The Netherlands.

[24]Department of Nuclear Medicine and Molecular Imaging, University Medical Center Groningen, Groningen, The Netherlands.

[25] Xing Imaging – A Mitro Company, London, UK

[26]Brain Sciences, Imperial College London, Hammersmith Hospital, London, UK.

[27]King's College London & Guy's and St Thomas' PET Centre, London, UK; School of Biomedical Engineering & Imaging Sciences, King's College London, London, UK.

[28]Department of Biomedical Engineering, New Jersey Institute of Technology, Newark, NJ, USA.

Supplementary Material

Running Title: Molecular Connectivity Nomenclature

# Correspondence to:

Prof. Rupert Lanzenberger, MD, PD

Email: rupert.lanzenberger@meduniwien.ac.at

ORCID: https://orcid.org/0000-0003-4641-9539

Medical University of Vienna, Department of Psychiatry and Psychotherapy, Austria

First Author	Year Published	Manuscript DOI	Tracer	Previously used Terminology	Proposed Terminology
Horwitz B.	1984	10.1038/jcbfm.1984.73	[^{18}F]FDG	rCMRglc correlations	Metabolic covariance
Horwitz B.	1985	10.1016/0166-4328(94)00139-7	[^{15}O]H20	Rate of cerebral blood flow correlations	Cerebral blood flow covariance
McIntosh A.R.	1993	10.1523/JNEUROSCI.14-02-00655.1994	[^{18}F]FDG	FDG connectivity	Metabolic covariance
Horwitz B.	1998	10.1073/pnas.95.15.8939	[^{15}O]H20	Rate of cerebral blood flow correlations	Cerebral blood flow covariance
Lee D.	2008	10.1007/s00259-008-0808-z	[^{18}F]FDG	Metabolic connectivity	Metabolic covariance
Hahn A.	2010	10.1523/JNEUROSCI.2409-10.2010	[*carbonyl*-^{11}C]WAY-100635	5-HT_{1A} receptor association	5-HT_{1A} covariance

Bose S.	2011	10.1038/npp.2011.113	[*carbonyl*-^{11}C]WAY-100635 and [^{11}C]DASB	5-HT$_{1A}$ and 5HT associations	5-HT$_{1A}$ and SERT covariance
Morbelli S.	2012	10.1016/j.neurobiolaging.2012.01.005	[^{18}F]FDG	Metabolic connectivity	Metabolic covariance
Di X.	2012	10.1089/brain.2012.0086	[^{18}F]FDG	Metabolic correlations/covariance	Metabolic covariance
Hahn A.	2014	10.1002/hbm.22442	[^{11}C]DASB	SERT associations	SERT covariance
Son S.	2015	10.1016/j.neures.2015.04.002	[^{18}F]FDG and [^{11}C]PIB	FDG-PET and PIB-PET connectivity	Metabolic and Amyloid beta covariance
Vanicek T.	2016	10.1038/srep28513	[^{18}F]FDG	Metabolic connectivity	Metabolic covariance
James GM.	2017	10.3389/fnhum.2017.00048	[^{11}C]DASB	SERT associations	SERT covariance

Vanicek T.	2017	10.1002/hbm.23418	[^{11}C]DASB	SERT associations	SERT covariance
Matheson G.J.	2017	10.1186/s13550-017-0304-1	[^{11}C]PBR28	TSPO correlations	TSPO covariance
Pereira J.	2017	doi: 10.1093/cercor/bhx294	[^{18}F]flortaucipir	[^{18}F]flortaucipir connectivity	Tau covariance
Veronese M.	2019	10.1038/s41598-019-39005-8	[^{18}F]FDG, [^{18}F]FDOPA and [^{11}C]SB217045	PET covariance	Metabolic covariance, Dopamine synthesis covariance and SERT covariance
Pillai R.	2019	10.1177/0271678X18764053	[*carbonyl*-^{11}C]WAY-100635	Molecular connectivity	5-HT$_{1A}$ covariance
Ossenkoppele R.	2019	10.1016/j.nicl.2019.101848	[^{18}F]flortaucipir	[^{18}F]flortaucipir covariance	Tau covariance

Iaccarino L.	2020	10.3233/JAD-190954	[^{18}F]FDG	Metabolic connectivity	Metabolic covariance
Verger A.	2020	https://doi.org/10.1007/s00259-019-04574-3	[^{18}F]FDG	Metabolic connectivity	Metabolic covariance
Jamadar S.	2021	10.1093/cercor/bhaa393	[^{18}F]FDG	Metabolic connectivity	Metabolic connectivity
Yakushev	2021	10.1007/s00259-021-05590-y	[^{18}F]FDG	[^{18}F]FDG covariance	Metabolic covariance
Reed MB.	2023	10.1016/j.neuroimage.2023.120030	[^{18}F]FDG	Metabolic connectivity	Metabolic connectivity
Volpi T.	2023	10.1177/0271678X231184365	[^{18}F]FDG	Metabolic connectivity	Metabolic connectivity
Wu G.	2023	10.1093/cercor/bhac286.	[^{18}F]FDG	Metabolic connectivity	Metabolic covariance
Wu G.	2023	10.1007/s00406-023-01637-3.	[^{18}F]FDG	Metabolic connectivity	Metabolic covariance

Vallini G.	2024	10.1007/s00259-024-06956-8	$[^{18}F]$FDG	Metabolic connectivity	Metabolic connectivity
Deery H.	2024	10.1038/s42003-024-07223-0	$[^{18}F]$FDG	Metabolic connectivity	Metabolic connectivity
Ruppert-Junck M.	2024	10.1007/s00259-024-06796-6	$[^{18}F]$FDG	Metabolic connectivity	Metabolic connectivity
Wu G.	2024	10.1038/s41398-024-03171-9	$[^{18}F]$FDG	Metabolic connectivity	Metabolic covariance

Supplementary table S1: **A selective overview of previous publications in the field of PET-based connectivtiy.** The previously used as well as the herein proposed terminology is provided. This highlights the use of numerous different terms for a single concept and proposes a clear terminology for future work. Please note that this table is not exhaustive but is intended as a collection of representative examples.

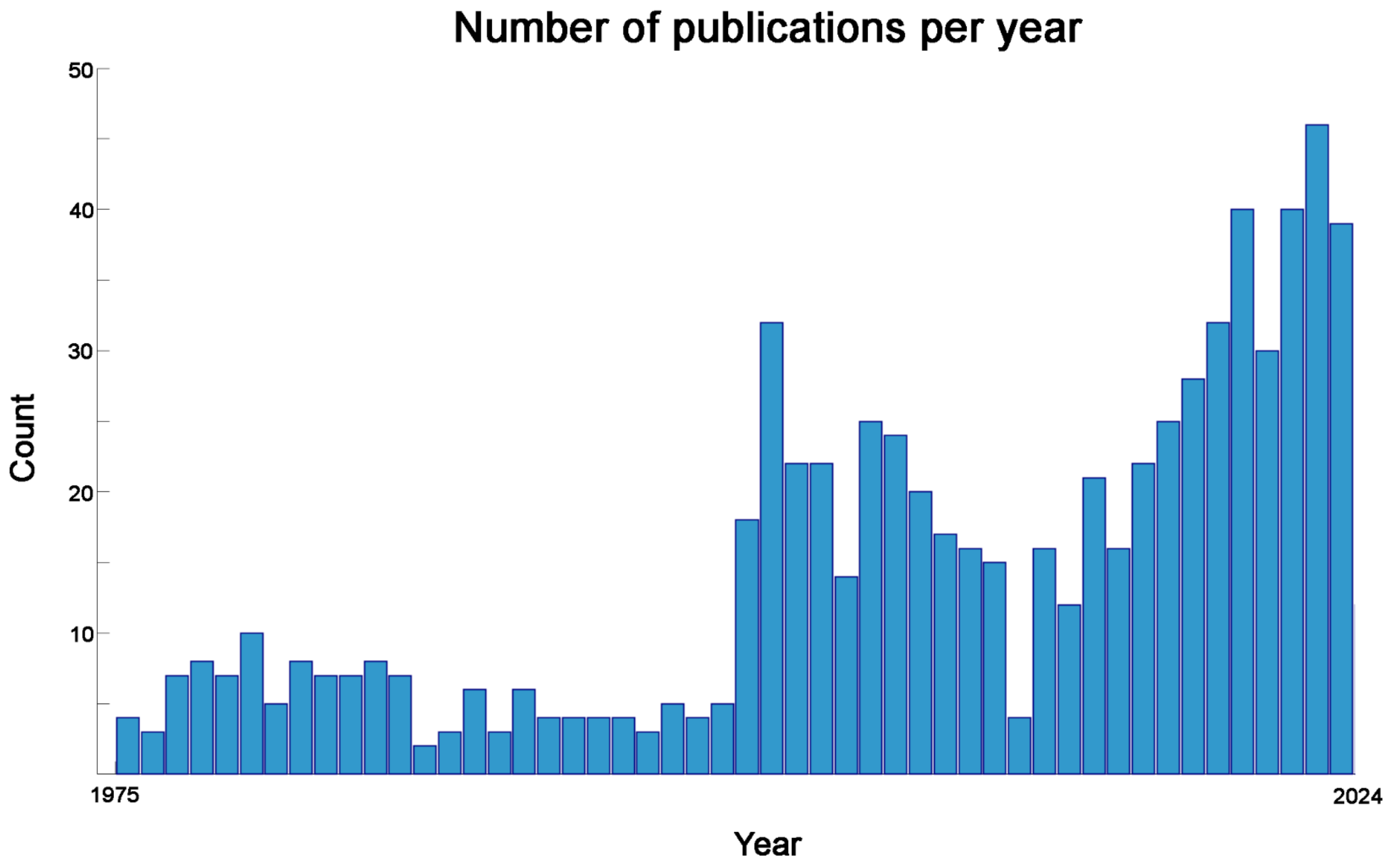

Supplementary Figure 1: Graphical overview of the number of publications related to molecular or metabolic connectivity from 1975 to 2024. The visualization shows an increasing amount of publications over the past 10 years. Pubmed search string was ("metabolic connectivity"[Title/Abstract] OR "molecular connectivity"[Title/Abstract]).
